# Exploring the double burden of malnutrition at the household level in the Philippines: Analysis of National Nutrition Survey data

**DOI:** 10.1371/journal.pone.0288402

**Published:** 2023-07-17

**Authors:** Josephine Gaupholm, Warren Dodd, Andrew Papadopoulos, Matthew Little

**Affiliations:** 1 Department of Population Medicine, University of Guelph, Guelph, Ontario, Canada; 2 School of Public Health Sciences, University of Waterloo, Waterloo, Ontario, Canada; 3 School of Public Health and Social Policy, University of Victoria, Victoria, British Colombia, Canada; International Institute of Tropical Agriculture (IITA), ZAMBIA

## Abstract

**Background:**

In the Philippines, the rising prevalence of obesity and related chronic diseases alongside persistent undernutrition presents a complex public health challenge. Understanding the patterns and dynamics of this ‘double burden of malnutrition’ (DBM) is crucial for developing effective intervention strategies. However, evidence of the occurrence of undernutrition and overnutrition within the same household is currently lacking.

**Methods:**

Using cross-sectional data from the 2013 Philippines National Nutrition Survey this study examined the prevalence of different typologies of household-level DBM from an analytical sample of 5,837 households and 25,417 individuals. Multivariable logistic regression was performed to identify factors associated with overall occurrence of intrahousehold DBM.

**Results:**

The overall prevalence of double burden households was 56% based on a comprehensive definition. The most common typology of intrahousehold DBM characterized in this study (% of all households) comprised households with at least one adult with overnutrition and at least one separate adult with undernutrition. Household size, wealth quintile, food insecurity, and household dietary diversity were all associated with household-level DBM. Double burden households were also influenced by head of household characteristics, including sex, level of education, employment status, and age.

**Conclusions:**

The findings from this study reveal that the coexistence of overnutrition and undernutrition at the household level is a major public health concern in the Philippines. Further comprehensive assessments of household-level manifestations of the DBM are needed to improve our understanding of the trends and drivers of this phenomenon in order to develop better targeted interventions.

## Introduction

The Philippines, like many low- and middle-income countries, is currently facing multiple burdens of malnutrition [1, 2]. Despite rapid economic growth, the Philippines continues to experience high levels of undernutrition (e.g., stunting, wasting, underweight) in addition to a rapid rise in the prevalence of overnutrition (e.g. overweight, obesity, and diet-related non-communicable diseases) [2–4]. Indeed, according to the 2022 Global Nutrition Report the prevalence of stunting among children under five is 28.8% in the Philippines, which is higher than the Asian regional average of 21.8% [2]. Meanwhile, approximately one third of adults are overweight or obese [2]. Micronutrient deficiencies also persist, with an estimated 70 to 80% of adults not meeting the recommended nutrient intakes for many vital micronutrients, including iron, calcium, and vitamin A [5]. This coexistence of both over- and undernutrition is known as the double burden of malnutrition (DBM) and can occur at the population, household, and/or individual level [6]. Understanding the patterns and dynamics of malnutrition is crucial for developing effective intervention strategies [7]. Studies of the DBM most frequently report on the coexistence of undernutrition and overnutrition at the population level [8]. DBM research in the Philippines is similarly population-level focused, with some recent work assessing individual-level DBM [9, 10]. However, household-level manifestations of the DBM have not been widely studied in the Philippines.

DBM at the household level is broadly characterized as the coexistence of undernutrition and overnutrition within the same household, however, no definitive operational definition or indicators of double burden households exist [8, 11]. While the majority of DBM research at the household level focuses solely on maternal-child relationships, where the presence of an overweight mother and a stunted child has largely become the archetypal characterization of a double burden household, numerous other typologies can, and do, exist [12–16]. In most studies, anthropometric measurements are used as indicators of undernutrition and overnutrition, however, exact indicators and cut-off points differ across studies [15]. As a result of such heterogeneity in DBM definitions and indicators, prevalence estimates of double burden households can vary widely between studies [8, 15, 16].

The cooccurrence of under- and overnutrition at the household level is often considered paradoxical, as historically these two forms of malnutrition were understood to arise from two different sets of determinants and behaviours [12, 17]. However, intrahousehold DBM requires that both under- and overnutrition arise among people who share similar food environments and household characteristics. As such, there is an increasing recognition that under- and overnutrition should not be considered distinct conditions at opposite ends of the nutrition spectrum, as both are indicative of malnutrition, including diets high in calories but low in micronutrients [6, 18]. Previous research has found associations between double burden households and socio-demographic factors, such as maternal age, educational level, and occupation, as well as household wealth and family size [11, 12, 14]. Suggested mechanisms driving intrahousehold DBM often relate to the nutrition transition and related changes in household dietary and lifestyle patterns [12]. Dietary behaviour and patterns are strongly influenced by social and familial dynamics. However, empirical evidence examining associations between intrahousehold DBM and dietary indicators, such as diet diversity, food security, and diet quality, is lacking [12, 19–21].

Understanding the various types and correlates of malnutrition at the household level provides an opportunity to identify shared drivers of malnutrition, which is critical for effectively designing and targeting interventions. However, in the Philippines, the extent and determinants of intrahousehold DBM are relatively unknown because of scarce information. To our knowledge, the only published research investigating household-level DBM in the Philippines reported that among a study sample of 376 child-mother pairs living in Metro Manila, 59% were experiencing dual forms of malnutrition [22]. However, since this localized study conducted two decades ago, no further examinations of household-level DBM have been done. To begin addressing this knowledge gap, the current study aims to explore this phenomenon using nationally representative data and gain a high-level understanding of factors associated with intrahousehold DBM. Specifically, the objectives of this study are: 1) to determine the prevalence of different typologies of the DBM at the household level in the Philippines; and 2) to examine the broad factors associated with the DBM at the household level.

## Methods

### Data source and study population

This study used publicly available data from the 2013 National Nutrition Survey (NNS) in the Philippines. The NNS, conducted by the Philippines Food and Nutrition Research Institute, is a cross-sectional, nationally representative survey that reports on the health, nutrition, and dietary composition of the Filipino population [23]. The 2013 NNS employed a three-stage stratified sampling design. Primary Sampling Units (PSUs) were defined first as a *barangay* (i.e., the smallest administrative unit in the Philippines) or contiguous *barangay* with at least 500 households. PSUs were then used to identify enumeration areas (EAs) of 150–200 households. A random selection of households within each EA made up the final sampling unit. Overall, 35,825 households and 172,323 individuals were sampled across 80 provinces (the province of Batanes was not surveyed due to logistic reasons). The complete methodology for the 2013 NNS survey has been detailed elsewhere [23, 24].

**Fig 1:**
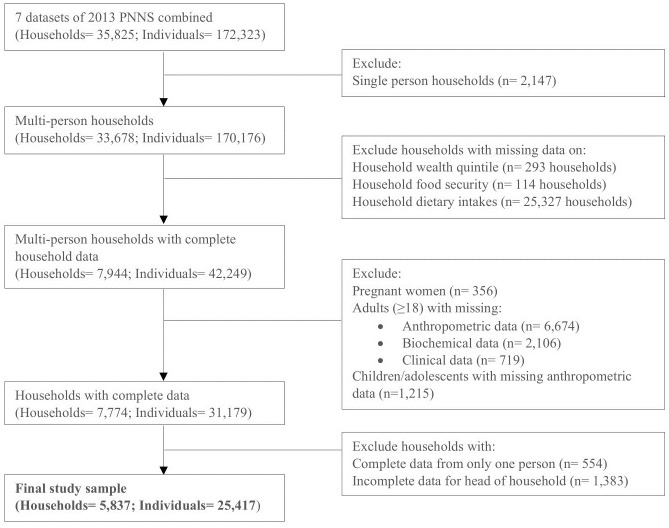
Flow diagram of exclusion criteria for both households and individuals used in this study (PNNS: Philippines National Nutrition Survey).

This study included multi-person households with complete data from seven survey components, including anthropometric, biochemical, clinical, food security, socioeconomic (household and individual), and dietary data. Data from pregnant women were excluded from the analysis because pregnancy affects the accuracy of anthropometric measurements [25]. A total of 5,837 households and 25,417 individuals were eligible for analysis (Fig 1).

### Outcome variables

#### Assessment of overnutrition and undernutrition at the individual level

Table 1 provides an overview of each of the indicators used to classify under- and overnutrition in adults and children and the clinical cut-offs. For adults, we used a combination of anthropometric, biochemical, and clinical indicators to classify malnutrition. Body mass index (BMI) was classified based on the World Health Organization (WHO) standard cut-offs [26]. Under- and overnutrition among children/adolescents were identified using height-for-age Z-scores (HAZ), weight-for-height Z-scores (WHZ), weight-for-age Z-scores (WAZ), and body-mass-index- for-age Z-scores (BAZ) [27]. Z-score calculations were performed using the Anthro and AnthroPlus macro packages for Stata developed by the WHO and United Nations Children’s Fund (UNICEF) which apply the WHO Child Growth Standards as reference data [28, 29]. Adults were classified as having overnutrition if they had any of the following conditions: (1) overweight/obesity or abdominal obesity; (2) hypertension; (3) hyperglycemia, or; (4) dyslipidemia. Adults were classified as having undernutrition if they had any of the following conditions: (1) underweight; (2) anemia; (3) vitamin A deficiency; or, (4) iodine deficiency. A child was considered under-nourished if they were stunted, wasted, and/or underweight. A child was considered over-nourished if they were overweight. Micronutrient deficiencies were not assessed for children and adolescents due to a limited number of complete micronutrient data from children and adolescents in our sample.

**Table 1:** Indicators used to classify undernutrition and overnutrition among the study sample.

	Indicator	Cut-off measurements
**Undernutrition (Children & adolescents)**	*Stunting*	HAZ ≤ -2 SD (≤17 years-old) [25]
	*Wasting*	WHZ ≤ -2 SD (<5 years-old) [25]
	*Underweight*	WAZ SD < -2 (≤10 years-old) [25]
**Overnutrition (Children & adolescents)**	*Overweight*	WHZ ≥ 2 SD (<5 years-old) [25]
		BAZ ≥ 2 SD (5 to 17 years-old) [25]
**Undernutrition (Adults ≥ 18 years-old)**	*Underweight*	BMI <18.5 kg/m^2^ [26]
	*Anemia*	Hb <12 g/dL [women] or <13 g/dL [men]* [30]
	*Vitamin A deficiency*	Serum retinol <10 μg/dL [31]
	*Iodine deficiency*	UIE <50 μg/L** [32]
**Overnutrition (Adults ≥ 18 years-old)**	*Overweight/obese*	BMI ≥25 kg/m^2^ [26]
	*Abdominal obesity*	WC ≥88 cm [women] or ≥102 cm [men] [33, 34]
	*Hyperglycemia*	Fasting blood glucose ≥110 mg/dl [35]
	*Hypertension*	Systolic BP ≥140 mmHg and/or diastolic BP ≥90 mmHg [34]
	*Dyslipidemia*	Triglycerides ≥150 mg/dL and/or HDL cholesterol <50 mg/dL [women] or <40 mg/dL [men] [36]

*Adjusted for smoking status. Hb concentrations for current smokers was reduced by 0.3 g/dL.

**Based on median urinary iodine concentrations.

Abbreviations; BAZ: body-mass-index- for-age Z-score; BMI: body mass index; BP: blood pressure; HAZ: height-for-age Z-score; Hb: hemoglobin; HDL: high-density lipoprotein; SD: standard deviation; UIE: urinary iodine excretion; WAZ: weight-for-age Z-score; WC: waist circumference; WHZ: weight-for-height Z-score

#### Anthropometric, biochemical, and clinical data collection

Height and weight measurements were obtained for all eligible household members using an electronic calibrated portable stadiometer (SECA 217, Hamburg, Germany) and a digital double window weighing scale (SECA 874, Hamburg, Germany). The recumbent length of children under 2 years-old was measured using an infantometer or a wooden length board. Waist circumference was measured among participants aged 10 and older using a calibrated tape measure. Blood pressure measurements were taken by trained nurses using a non-mercurial sphygmomanometer (A&D Um-101TM) and stethoscope. Registered medical technologists collected blood samples which were used to measure biomarkers of cardio-metabolic risk factors (CMRF), namely fasting blood glucose and blood lipids. Following collection, they were analyzed using the enzymatic colorimetric method with Roche COBAS Integra and Hitachi 912. Registered medical technologists also collected blood and urine samples to assess micronutrient deficiencies. The cyanmethemoglobin method was used to evaluate hemoglobin levels. Serum retinol was examined using High-Performance Liquid Chromatography. Urinary iodine excretion levels were determined using the acid digestion/colorimetric method.

#### Assessment of the double burden of malnutrition at the household level

To comprehensively evaluate the prevalence of double burden households in the Philippines, we considered all possible typologies, or combinations, of under- and overnutrition among household members. A household was considered a double burden household if they had any combination of undernutrition and overnutrition in two or more household members (Table 2). We also explored the prevalence of several specific household DBM subtypes relevant to the literature, including the co-occurrence of child undernutrition and maternal overnutrition (represented by women of reproductive age 18–49 years).

**Table 2:** Definitions used to define the various types of household-level double burden of malnutrition.

DBM type	Definition
**Overall**	Coexistence of undernutrition and overnutrition within the same household
**1**	An adult with overnutrition and a child/adolescent with undernutrition
**1.1**	A woman of reproductive age (18 to 49 y) with overnutrition and a child/adolescent with undernutrition
**1.2**	A woman of reproductive age (18 to 49 y) with overnutrition and a child under 5 with stunting and/or wasting
**2**	Adult with undernutrition and child/adolescent with overnutrition
**3**	An adult with overnutrition and a separate adult with undernutrition
**3.1**	An adult with overnutrition and a separate adult with undernutrition, but no children/adolescents with malnutrition
**3.2**	An adult man (≥18 y) with overnutrition and an adult woman (≥18 y) with undernutrition
**3.3**	An adult woman (≥18 y) with overnutrition and an adult man (≥18 y) with undernutrition
**4**	A child/adolescent with overnutrition and a separate child/adolescent with undernutrition
**5**	Households with all forms of malnutrition (adult with overnutrition, adult with undernutrition, a child/adolescent with undernutrition, and a child/adolescent with overnutrition)

### Independent variables

Covariates were identified from previous literature and included both household factors and individual-level characteristics of the head of household [11–14]. These include the age, sex, educational attainment, and occupation of the head of household. Household factors included the number of people living in the household (household size), wealth quintile, food security, and diet diversity.

The head of household was reported by participants during the survey. The sex of the head of household was classified as either male or female, while the age variable was categorized as 18–29, 30–39, 40–49, 50–64, and ≥65. Educational attainment was classified as either having a post-secondary education or not. Household size was categorized into four groups: 2–3, 4–6, 7–10, and ≥11. Wealth status was derived from principal component analysis scores based on household assets such as possession of vehicles, appliances, housing construction materials, and sanitation facilities [23]. These scores were used to define wealth quintiles.

The Household Food Insecurity Access Scale (HFIAS) was used to assess household food security. The nine-question HFIAS measures the occurrence and frequency of various conditions related to food insecurity over a one-month (30 day) period. Participants are first asked an occurrence question—i.e., whether a condition occurred at all in the past 30 days (yes or no). If they respond yes, they are then asked the frequency-of-occurrence, that is, if the condition happened rarely (once or twice), sometimes (three to ten times) or often (more than ten times) in the past four weeks. Using the scoring system developed by USAID’s Food and Nutrition Technical Assistance Project [37], households were categorized as either “food secure”, “mildly food insecure”, “moderately food insecure”, or “severely food insecure”.

Household diet diversity scores (HDDS) were calculated based on data collected through one-day food records, which including weighing foods consumed inside the home. Trained registered nutritionist-dietitians used a digital dietetic scale to weigh (before cooking or serving) all food items prepared and served in the home for an entire day. Unconsumed or leftover food was deducted from the total in order to obtain the final amount of food and beverages consumed by the household. Foods consumed outside the house were recalled by household members and added to the household food record. Dietary data were aggregated into 12 food groups based on Food and Agriculture Organization guidelines [38]. HDDS were computed by summing up the total number of food groups consumed by members of the household. Scores were then categorized into three groups representing low diet diversity (score of 1 to 4), moderate diet diversity (5 to 7), and high diet diversity (≥8).

### Statistical analysis

We generated descriptive statistics for the socio-demographic and individual over- and undernutrition characteristics of the study sample using weighted means and percentages. Survey sample weights were computed and adjusted for non-response, then post-stratified based on the projected population obtained from the Philippines Statistics Authority. We calculated the prevalence of all possible combinations of overnutrition and undernutrition within households, with overall presence being categorized as a binary outcome (0 = not a double burden household, 1 = a double burden household). The rationale for this approach was based on the desire to gain a high-level understanding of factors associated with the phenomenon of intrahousehold DBM in general across the Philippines, as this has not yet been explored in the literature. Additionally, following preliminary explorations of the data, we found that assessing all possible typologies of household DBM separately increased the risk of misclassification bias and diminished the statistical power of the analyses. It was also for this reason that micronutrient deficiencies were not assessed separately from underweight.

Logistic regression analyses were used to examine factors associated with double burden households of any type. First, bivariate regression models were constructed to examine the unconditional association between each of the independent variables and our dependent variable (i.e., household-level DBM). As we had identified our variables of interest *a priori* based on previous research, all covariates, regardless of significance in the unconditional analysis, were included in the multivariable logistic regression model. Prior to fitting our main model, we examined all pairwise correlations among predictor variables to test for possible collinearity using Spearman’s rho. However, we found no evidence of multicollinearity between independent variables as all correlation coefficients were less than |0.80|. As such, a model containing all variables of interest was constructed. This model also included dummy variables for the 17 administrative regions in the Philippines to control for geographic differences. Subsequently, we tested for all plausible two-way interactions among the covariates included in our main model, however, none were found to be significant. Goodness of fit was assessed using the Hosmer-Lemeshow goodness of fit test for binary data. All statistical analyses were performed using the Stata statistical software package version 16.1 (2022; StataCorp). The level of significance was set at p<0.05.

## Results

### Individual nutrition status among study participants

We analysed the individual nutrition status of 13,843 adults (≥18 y) and 11,574 children/adolescents (≤17 y). The mean BMI of adults in the sample was 23.06 kg/m^2^ (SD = 4.18), with 31.2% of women and 26.2% of men being either overweight or obese (Table 3). Dyslipidemia was the most prevalent CMRF, affecting nearly one in three adults within the study sample. The prevalence of abdominal obesity was notably 6.8 times higher in women than in men (20.5% compared to 3%). Overall, 55% of adults had one or more indicator of overnutrition. Conversely, undernutrition affected 41.7% of adults. Approximately 11% of adults were underweight (BMI <18.5 kg/m^2^), while over one in three adults had some form of micronutrient deficiency.

**Table 3:** Prevalence of individual malnutrition indicators among adults *(*≥18 years old) in the Philippines in 2013.

	Overall (n = 13,843)		Adult Women (n = 6,771)		Adult Men (n = 7,072)	
Malnutrition Status	Mean (SD)	%	Mean (SD)	%	Mean (SD)	%
BMI (kg/m^2^)	23.06 (4.18)		23.30 (4.45)		22.83 (3.90)	
*Underweight (<18*.*5 kg/m*^*2*^*)*		11.17		12.11		10.28
*Normal (18*.*5–24*.*9 kg/m*^*2*^*)*		60.17		56.67		63.53
*Overweight (25*.*0–29*.*9 kg/m*^*2*^*)*		22.92		24.07		21.82
*Obese (≥30*.*0 kg/m*^*2*^*)*		5.73		7.15		4.37
Waist Circumference (cm)	79.02 (11.27)		78.40 (11.54)		79.61 (10.97)	
*Abdominal obesity (≥88 cm women; ≥102 cm men)*		11.57		20.51		3.03
Hypertension (BP ≥140/90 mmHg)		9.73		8.76		10.67
Dyslipidemia (Triglycerides ≥150 mg/dL and/or HDL <50 mg/dL women; <40 mg/dL men)		32.48		28.51		36.31
Hyperglycemia (FBG ≥110 mg/dl)		10.23		9.31		11.13
Anemia* (HB <12 g/dL women; <13 g/dL men)		11.84		14.15		9.61
Vitamin A deficiency (Serum retinol <10 μg/dL)		0.11		0.14		0.08
Iodine deficiency (UIE <50 μg/dL)	99**	24.23	91**	26.79	107**	21.90

* Adjusted for smoking status

** Median values

Based on 2013 Philippines National Nutrition Survey data.

Table presents weighted means and percentages.

Abbreviations; BP: blood pressure; FBG: fasting blood glucose; HB: hemoglobin; HDL: high-density lipoprotein; UIE: urinary iodine excretion

Table 4 presents the prevalence of malnutrition among children and adolescents. Overall, over one in three children and adolescents were classified as having some form of undernutrition, while only 3.3% were considered overweight. Stunting was the most prevalent form of undernutrition, affecting nearly 30% of all children/adolescents.

**Table 4:** Prevalence of individual malnutrition indicators among children and adolescents (≤17 years old) in the Philippines 2013 (n = 11,574).

Malnutrition Status	Age group	Overall %	Female %	Male %
Stunting	*All*	28.36	26.19	30.47
	*<5 y*	26.67	25.28	28.09
	*5–10 y*	27.82	25.67	29.81
	*11–17 y*	30.00	27.22	32.63
Wasting	*<5 y*	7.60	8.31	6.87
	*5–10 y*	-	-	-
	*11–17 y*	-	-	-
Underweight	*All*	23.99	23.79	24.20
	*<5 y*	18.78	20.42	17.10
	*5–10 y*	29.89	27.75	31.93
	*11-17y*	-	-	-
Overweight	*All*	3.30	2.32	4.24
	*<5 y*	4.54	3.69	5.41
	*5–10 y*	3.52	1.84	5.11
	*11–17 y*	2.21	1.58	2.82

Based on 2013 Philippines National Nutrition Survey data.

Table presents weighted percentages.

### Household-level double burden

The overall prevalence of double burden households was 56% when we included all possible combinations of over- and undernutrition among household members (Table 5). The majority of these double burden households (83.3%) had at least one adult with overnutrition and at least one adult with undernutrition. The proportion of households with DBM type 1, i.e., at least one adult with overnutrition and at least one child/adolescent with undernutrition, was 26.8%. When we restricted DBM type 1 to only include the co-existence of overnutrition among women of reproductive age (18–49 y) and undernutrition among children/adolescents, the prevalence of DBM fell to 12%. Further, if we define child undernutrition using only stunting and wasting metrics among children under five, household-level DBM prevalence was 4.3%.

**Table 5:** Prevalence of the various types of the household-level double burden of malnutrition in the Philippines based on 2013 National Nutrition Survey data.

DBM type	Definition	n(%)
**Overall**	Coexistence of undernutrition and overnutrition within the same household	3,247(55.63)
**1**	Adult with overnutrition and child/adolescent with undernutrition	1,562(26.76)
**1.1**	A woman of reproductive age (18–49 y) with overnutrition and a child/adolescent with undernutrition	673(11.53)
**1.2**	A woman of reproductive age (18–49 y) with overnutrition and a child under 5 with stunting and/or wasting	251(4.30)
**2**	Adult with undernutrition and child/adolescent with overnutrition	188(3.22)
**3**	An adult with overnutrition and a separate adult with undernutrition	2,706(46.36)
**3.1**	An adult with overnutrition and a separate adult with undernutrition, but no children with malnutrition	1,549(26.54)
**3.2**	An adult man with overnutrition and an adult woman with undernutrition	1,467(25.13)
**3.3**	An adult woman with overnutrition and an adult man with undernutrition	1,224(20.97)
**4**	A child/adolescent with overnutrition and a separate child/adolescent with undernutrition	101(1.73)
**5**	Households with all forms of malnutrition (an adult with overnutrition, an adult with undernutrition, a child/adolescent with undernutrition, and a child/adolescent with overnutrition)	57(0.98)

### Socio-demographic characteristics

Table 6 describes the sociodemographic characteristics of the study population overall and by household DBM type. Overall, households had on average five members, with the majority having a male head of household. Households were relatively evenly distributed across each of the wealth quintiles, however, close to 60% of households were classified as either moderately or severely food insecure. Household heads were mainly employed as farmers, laborers, or unskilled workers, with only 15.7% having attended postsecondary school.

**Table 6:** Characteristics of sample households overall and by household DBM type, from the 2013 Philippines National Nutrition Survey (n = 5,837).

	All households (n = 5,837)	Non-DBM households (n = 2,590)	All DBM households (n = 3,247)	DBM Type 1 households (n = 1,562)	DBM Type 2 households (n = 188)	DBM Type 3 households (n = 2,706)	DBM Type 4 households (n = 101)
**Head of household characteristics**	Mean ± SD/percentage (%)	Mean ± SD/percentage (%)	Mean ± SD/percentage (%)	Mean ± SD/percentage (%)	Mean ± SD/percentage (%)	Mean ± SD/percentage (%)	Mean ± SD/percentage (%)
Sex							
*Male*	83.66	82.26	84.84	86.45	79.00	84.64	80.34
*Female*	16.34	17.74	15.16	13.55	21.00	15.36	19.66
Age	49.92 ± 12.86	48.18 12.69	51.38 ± 12.82	48.19 ± 11.99	51.96 ± 12.79	52.70 ± 12.73	49.54 ± 13.44
Employed							
*Yes*	84.22	86.41	82.39	85.71	77.25	81.27	83.76
Occupation							
*Farming/laborers/unskilled work*	42.48	43.03	42.01	46.01	31.45	41.65	37.16
*Factory/trades*	19.20	19.08	19.30	21.66	15.97	18.72	17.52
*Service/sales*	6.00	6.64	5.45	4.64	5.78	5.54	8.25
*Professional/government work*	12.05	12.42	11.73	9.99	16.46	11.24	17.45
*Housekeeper*, *Pensioner*, *Student*	8.29	8.56	8.07	5.03	14.79	8.88	8.95
*Unemployed*	11.99	10.27	13.43	12.68	15.53	13.97	10.68
Education level							
*Postsecondary education*	15.71	17.67	14.08	11.00	23.80	14.45	16.69
*No postsecondary education*	84.29	82.33	85.92	89.00	76.20	85.55	83.31
**Household characteristics**							
Household size	5.39 ± 2.30	4.92 2.04	5.79 ± 2.42	6.55 ± 2.44	7.02 ± 2.72	5.72 ± 2.47	7.81 ± 2.83
Wealth quintile							
*1*	20.48	22.26	19.06	25.74	11.07	18.07	23.26
*2*	21.62	21.43	21.76	24.10	10.74	21.52	13.04
*3*	20.85	19.80	21.70	21.38	17.66	21.75	19.42
*4*	19.34	18.69	19.86	17.94	22.32	20.36	18.73
*5*	17.71	17.82	17.62	10.85	38.21	18.29	25.55
Household food insecurity (access) category							
*Food secure*	26.53	29.06	24.50	18.19	35.65	25.50	23.19
*Mildly food insecure*	14.45	13.62	15.11	12.99	15.67	15.48	11.96
*Moderately food insecure*	38.29	36.80	39.49	41.78	29.27	38.51	31.15
*Severely food insecure*	20.73	20.52	20.90	27.05	19.41	20.51	33.70
Household diet diversity score							
*1–4*	5.11	6.07	4.34	5.40	1.63	4.05	2.84
*5–7*	41.31	43.34	39.69	40.79	23.75	40.35	28.71
*≥8*	53.57	50.60	55.97	53.80	74.63	55.60	68.45

Table presents weighted means and percentages.

Abbreviations; DBM: double burden of malnutrition; SD: standard deviation

### Factors associated with household-level double burden

Table 7 presents the results from both our bivariate and multivariable logistic regression models, reporting the unadjusted odds ratios (uOR) and adjusted odds ratios (aOR), respectively. The remainder of the results section focuses on the results of the multivariable analysis. Female-headed households had significantly lower odds of household-level DBM compared to male-headed households (95% confidence interval (CI): 0.63–0.86). Similarly, the odds of being a double burden household were lower when the head of household attended post-secondary school (95% CI: 0.63–0.89). The age of the head of the household was positively associated with the household experiencing a DBM. For example, intrahousehold DBM was 2.22 times more likely in households with a head of household aged 65 or older, compared to households with a head of household between the ages of 18 to 29 (95% CI: 1.51–3.14). We found no significant differences in household-level DBM between head of households employed as farmers, laborers, or in unskilled work and other occupation categories. However, households with an unemployed head of household were found to have 1.24 times higher odds of household-level DBM compared to household heads employed in farming, labour, or unskilled work (95% CI: 1.03–1.49).

**Table 7:** Results from bivariate and multivariable logistic regression models showing factors associated with the overall double burden of malnutrition at the household-level in the Philippines, 2013 (n = 5,837).

Variable	Bivariate models			Multivariable model		
	uOR^a^	95% CI	P-value	aOR^b^	95% CI	P-value
Head of household characteristics						
Sex						
*[Male]*						
*Female*	**0.80** ***	0.70–0.92	0.001	**0.73** ***	0.63–0.86	<0.001
Age category						
***[18–29]***						
*30–39*	1.12	0.79–1.58	0.539	0.95	0.66–1.36	0.772
*40–49*	**1.49** *	1.06–2.08	0.021	1.18	0.83–1.66	0.367
*50–64*	**1.94** ***	1.39–2.71	<0.001	**1.67** **	1.18–2.35	0.004
*≥65*	**2.05** ***	1.45–2.90	<0.001	**2.22** ***	1.51–3.14	<0.001
Employment						
*[Farming/laborers/unskilled work]*						
*Factory/trades*	1.07	0.92–1.24	0.389	1.07	0.91–1.26	0.405
*Service/sales*	0.85	0.67–1.08	0.173	0.99	0.76–1.28	0.946
*Professional/government work*	0.97	0.81–1.15	0.709	1.12	0.91–1.37	0.276
*Housekeeper/pensioner/student*	0.92	0.76–1.10	0.362	0.91	0.73–1.14	0.427
*Unemployed*	**1.34** ***	1.13–1.58	0.001	**1.24** *	1.03–1.49	0.022
Education level						
*[No postsecondary]*						
*Postsecondary education*	**0.77** ***	0.67–0.89	<0.001	**0.75** ***	0.63–0.89	0.001
Household characteristics						
Household size category						
***[2–3]***						
*4–6*	**1.39** ***	1.22–1.59	<0.001	**1.49** ***	1.29–1.71	<0.001
*7–10*	**2.45** ***	2.09–2.87	<0.001	**2.56** ***	2.16–3.03	<0.001
*≥11*	**4.90** ***	3.33–7.20	<0.001	**4.82** ***	3.24–7.16	<0.001
Wealth quintile						
***[1]***						
*2*	**1.18** *	1.01–1.37	0.034	1.15	0.98–1.35	0.094
*3*	**1.30** ***	1.11–1.52	0.001	**1.25** **	1.05–1.49	0.013
*4*	**1.22** *	1.03–1.43	0.018	1.20	0.99–1.46	0.065
*5*	1.18	0.99–1.40	0.058	**1.26** *	1.00–1.59	0.049
Household diet diversity score						
***[0–4]***						
*5–7*	**1.29** *	1.03–1.62	0.026	1.27	1.00–1.61	0.053
*≥8*	**1.53** ***	1.22–1.91	<0.001	**1.46** **	1.14–1.87	0.003
Household food insecurity (access) category						
*[Food secure]*						
*Mildly food insecure*	**1.24** *	1.05–1.47	0.013	**1.23** *	1.03–1.48	0.025
*Moderately food insecure*	**1.21** **	1.06–1.38	0.005	**1.23** **	1.06–1.44	0.008
*Severely food insecure*	**1.18** *	1.01–1.37	0.033	**1.20** *	1.00–1.44	0.050

^a^ Results from the unadjusted (bivariate) logistic regression models.

^b^ Results from the multivariable model, adjusted for all variables of interest as well as geographic region (values not shown).

Abbreviations; OR: odds ratio; CI: confidence interval

Items in the square brackets [] represent the referent category

*p≤0.05

**p≤0.01

***p≤0.001

Larger household size was associated with higher odds of household-level DBM. Additionally, households experiencing food insecurity had higher odds of intrahousehold DBM compared to food-secure households. Household wealth quintile was also associated with household DBM, with households in the 5^th^ (or wealthiest) quintile having 1.26 times the odds of household-level DBM when compared to the 1^st^ (or lowest) quintile (95% CI: 1.00–1.59). Lastly, higher household diet diversity scores were associated with increased odds of household-level DBM. For instance, households with high household diet diversity (i.e., a HDDS of 8 or above) had 1.46 times the odds of household DBM when compared to households with low diet diversity (i.e., a HDDS of 4 or below) (95% CI: 1.14–1.87).

## Discussion

The current study sought to evaluate the prevalence, typologies, and correlates of the household-level DBM in the Philippines. Using data from a large nationally representative survey, we adopted a comprehensive definition of household-level DBM to examine this complex phenomenon. Results indicate that various types of household-level DBM exist in the Philippines, with over half of all households experiencing some form of intrahousehold DBM. This reflects both the high prevalence of individual-level indicators of over- and undernutrition among the Filipino population, but also the number and type of individual-level indicators we measured which included anthropometric measurements, micronutrient deficiencies, and CMRF. The growing concern of overweight and associated CMRF in the Philippines was emphasized in this study, as over one in two adults were affected by some form of overnutrition. On the other hand, undernutrition remains a serious public health concern in the Philippines, with 41.7% of adults and 33.5% of children and adolescents exhibiting at least one indicator of undernutrition. At the household-level, these forms of malnutrition manifested in a myriad of ways. The prevalence of households containing a woman of reproductive age (18–49 years) with overnutrition and a child with undernutrition, which is the most frequently assessed household-level DBM type in the literature, was 12%. While this figure is comparable to the global average prevalence, there is large variation between studies, regions, and countries [6, 11]. A recent systematic review by Kosaka and Umezaki found that household prevalence of mother overweight–child underweight pairs ranged from <3% (e.g., in Bangladesh, Cambodia, and Nepal) to >10% (e.g., in Bolivia, Egypt, and Guatemala) [11]. Of the household-level DBM typologies characterized in this study, the most common (46.4% of all households) comprised households with at least one adult with overnutrition and at least one different adult with undernutrition. Of these pairings, 54.2% (or 25% of all households) consisted of a man (≥18 years) with overnutrition and a woman (≥18 years) with undernutrition. The high prevalence of this pairing is notable, as there is a substantial lack of research exploring such adult-adult DBM parings in the literature [8]. Previous studies on DBM may have overlooked this important manifestation of household-level DBM. This typology of DBM may be indicative of inequitable allocation of resources (e.g., food, financial resources, and decision-making power) among adults at the household level, which bears further investigation.

Our analysis identified a number of socio-economic and demographic factors associated with intrahousehold DBM. Consistent with previous research, we found positive associations with both the age of the head of household and household size [11]. Household wealth was also associated with household-level DBM, with households in the 3^rd^ and 5^th^ wealth quintiles having significantly higher odds of household-level DBM compared to households in the 1^st^ (or lowest) wealth quintile. This indicates that middle- and high-income households may be at higher risk for intrahousehold DBM in the Philippines, which supports findings from similar research [14, 39].

Our results showed female-headed households had lower odds of DBM compared to male-headed households. Correspondingly, a similar study using nationally-representative survey data from Indonesia found that female-headed households were less likely to experience the DBM, defined as households with at least one overweight and one underweight member [40]. This association underscores the importance of gender dynamics and decision-making power in household nutrition. Evidence suggests that women’s empowerment in the household, including decision-making power and autonomy, is associated with improved child nutrition in Asia [41], Africa [42], and other regions [43]. It has been hypothesized that when women have greater control and access to household resources, they tend to prioritize health and food spending, therefore leading to improved diets and nutrition [40]. However, such relationships are complicated by the reality that female-headed households are at increased risk of food insecurity and poverty [44] due to numerous economic and societal disadvantages, including unequal access to assets (e.g., land and livestock), markets, and services [45, 46]. Indeed, contrary to our findings, Sansón-Rosas et al. found that female-headed households in rural Colombia had higher odds of intrahousehold DBM [47], while results from a pooled analysis of 23 African countries found no association with the sex of the head of household [14]. Conflicting results across studies and contexts could be influenced by the variation in sample populations, study design, and/or measures of household DBM.

It has been previously reported that the Philippines has one of the highest burdens of food insecurity in southeast Asia [48]. Food insecurity is strongly associated with a number of nutritional and cardio-metabolic health outcomes, including indicators of both under- and overnutrition [49]. In our analysis, food insecurity was associated with increased odds of household-level DBM, indicating that food insecurity may reduce household dietary quality and increases risk of multiple forms of malnutrition. Households experiencing food insecurity often modify their diets by decreasing the overall amount of food consumed and increasing consumption of cheaper, energy-dense, highly processed foods, thus creating a nutrition environment in which multiple forms of malnutrition can develop [48]. Studies from Brazil, Indonesia, and Colombia also found food insecurity was associated with the DBM at the household level [47, 50, 51]. Further research is needed to examine the precise mechanisms for this association, including how food insecurity interacts with environmental (e.g., food environment) and household (e.g., intrahousehold allocation of resources) factors to modify dietary consumption and influence risk of nutritional health outcomes [52].

We also found that household dietary diversity scores were associated with increased odds of intrahousehold DBM. This finding is contrary to our expectations, as the HDDS is often viewed as an indicator of a household’s economic ability to access food [38] and diversity is often seen as a key component of healthy diets [53]. While the HDDS is not intended as a proxy for dietary quality [38], studies elsewhere have established a positive association between household diet diversity and healthy diets [54], food security [55], and nutrition outcomes [56]. One might therefore expect that households with higher HDDS would have lower odds of DBM; however, we observed the inverse association in our analysis. This association was consistent across bivariate analyses and when controlling for household food security, wealth status, and occupation in the multivariable model. This finding may indicate that in the Philippines, households with greater dietary diversity consume larger quantities of non-nutrient-dense foods and/or live more sedentary lifestyles. Another possibility is the presence of intrahousehold disparities in resource allocation, leading to nutritional inequalities that are not identified by the household-level HDDS. However, such explanations remain purely conjecture given the limited information provided by HDDS. These findings may raise questions about the HDDS as an accurate indicator of food access and dietary quality. Researchers have previously criticized dietary diversity scores for their limited cross-cultural validity and misuse/misinterpretation in nutrition literature [57–59]. Other authors have critiqued the reliability and usefulness of the HDDS [57, 60] as a research tool and promote tools (e.g., the World Food Programme’s Food Consumption Score) that assign higher weights to foods deemed most important for nutritional purposes [61]. Further research is therefore required to ensure validity and reliability of the HDDS across multiple global contexts [60], particularly since improved dietary diversity is often measured in DBM research [14] and is a stated goal of nutrition interventions. Future studies examining dietary determinants of intrahousehold DBM should also focus on measuring household dietary intake and quality, not just diversity.

This research provides important and novel insights into the household-level double burden of malnutrition in the Philippines. However, our study has several limitations. First, although we use data from a large, nationally representative sample, we were limited to the information and variables collected by the NNS. For instance, it was not possible to establish familial relations within each household (for example, in households with children and multiple women of reproductive age, we could not definitively identify mother-child pairs), which may limit the comparability of results with research on household-level DBM elsewhere. Second, while this the most recent NNS with publicly available data, the situation in the Philippines today has likely changed since 2013. Similar analysis of future NNS data should be done to get a more accurate picture of the intrahousehold DBM in the Philippines. Third, due to our use of a comprehensive definition of household-level DBM rather than focusing on the more common mother-child DBM pairs, the comparability of our study to other research on household-level DBM is limited. Further, although we did include some indicators of micronutrient deficiencies among adults, this paper did not assess the presence of these conditions as separate from underweight, or what is known as the triple burden of malnutrition. Due to limitations of the data set, which lacked an adequate number of complete micronutrient data from children and adolescents in our sample, assessing triple burden and double burden typologies separately increased the risk of misclassification bias and diminished the statistical power of the analyses.

In conclusion, we found a very high prevalence of intrahousehold DBM when considering all possible typologies of under- and overnutrition among household members in the Philippines, indicating that this form of the double burden of malnutrition is a major public health concern. This study highlights important gaps in the current understanding of household-level manifestations of the DBM, particularly those between adult-adult DBM parings. While further research is needed to investigate each of these specific typologies in greater detail, these findings support the need for more comprehensive surveillance of the DBM at the individual, household, and national levels in order to monitor progress and develop better targeted interventions. We identified a number of socio-economic and demographic factors associated with overall intrahousehold DBM, including household size, wealth quintile, food insecurity, and diet diversity. Double burden households were also influenced by head of household characteristics including sex, level of education, employment status, and age. These results suggest that key policy actions for reducing the DBM should center on supporting education and employment opportunities, gender equality, and social safety nets. Overall, this evidence provides important high-level insight into factors influencing household-level DBM in the Philippines and may help inform future research designs and directions.
